# Supplementary material for: Learning curve and surgical outcome of robotic assisted colorectal surgery with ERAS program
Source: Sci Rep. 2022 Nov 29;12:20566. doi: 10.1038/s41598-022-24665-w (PMC9709162; doi:10.1038/s41598-022-24665-w)
Supplement: Supplementary file 1 — Supplementary Information 1. [file 41598_2022_24665_MOESM1_ESM.zip › LSC Raw data-Ñ[▒K/050/OP N_ñw╝╨░O▒Kñσ.pdf]

W95 / W95

臺中榮民總醫院
手術室護理記錄

[redacted]

基本資料

- 室別： 開刀房-06室，手術科別： CRS，手術類別： 常規，日期： 2019/10/15
- 進入時間： 1905 ； 麻醉開始時間： 1905 ； Time Out時間： 2045 ； 劃刀時間： 2046 ； 手術結束時間： 0300 送出時間： 0310 ；
- 麻醉方式： GE
  診斷：rectum ca 2 cm FAV s/p CCRT. TaTME+ LLND+ 3D.
  手術名稱：1.Low anterior resection with colo-anal anastomosis
  2. Lymphadenectomy, pelvic
  3.Ileostomy
  4. Technical fee of 3D complex minimally invasive surgery≧5 hrs
  手術局部用藥：nil
  術前預防性抗生素：
    - 抗生素名稱一： Veterin 1GM IVD由麻醉護理師給予
  ，追加預防性抗生素：
    - 抗生素名稱一： Veterin 1GM IVD由麻醉護理師給予
    - 時間： 0020
    - 上次簽章: 徐敬家
- 手術醫師： 林俊余，江梓維，黃俋霖，陳映菁，林陳彥，林宜慧
- 麻醉醫師： VS 張詒婷
-
    - 刷手護士A ： 陳心慈，時間： 1905，接替刷手一： 李苓瑜，時間： 2000，接替刷手二： 羅渝軒，時間： 2300
    - 流動護士A ： 林怡廷，時間： 1905，接替流動一： 許臺真，時間： 2000，接替流動二： 徐敬家，時間： 2300

皮膚狀況

A-V Shunt： 無
術前： 完整
記錄者： 上次簽章: 林怡廷
術後： 完整
記錄者： 上次簽章: 徐敬家

手術準備

手術臥式 單一手術臥式

-
    - 手術臥式： 截石臥式
    - 使用附件： 溫毯 38 ℃，矽膠枕頭，腳架，其它： 頭架
    - 皮膚準備： 10%Aq Betadine，Hibiscrub，其它： 2% CHLORHEXIDINE
    - 電燒： 使用電燒: 電極片位置： 右大腿，右腹部，記錄者： 上次簽章: 許臺真

止血帶T： 無

植入物

- 植入物名稱/號碼/數量/部位：GIA80mm-3.8mm*1/直腸斷端

, 記錄者：

標本送檢

- 細菌培養 無
- 細胞學 無
- 冰凍切片 無
- 病理標本
  組織名稱一：RECTUM
  ,

組織名稱二： IMA ROOT

,

組織名稱三： R'T PELVIC LN

- 截肢 無

標本交付 標本： 3 袋，標本： 0 盒，標本： 0 瓶，細胞學： 0 管，Culture Tube： 0 管，被交付醫師： CR 黃俋霖，

- 記錄者： 上次簽章: 徐敬家
- 時間： 0300

傷口術後敷料

傷口敷料： 傷口敷料： 無

- 請於圖上標示部位：

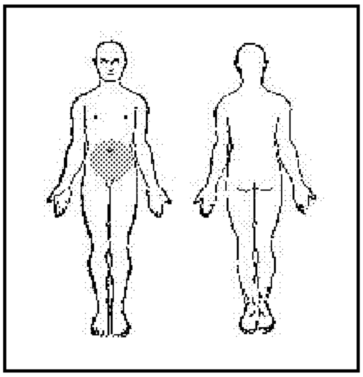

- 傷口敷料： guaze

導管

導管	天數	導管資訊	插入日期	插入時間	更換日期	拔除日期	拔除時間	拔除原因	預計拔除/更換	預計拔除/更換日期
Foley(1)	2	號碼:16 Fr.; 部位:尿道;	2019/10/15	1900						
Sump NG Tube(1)	2	號碼:16 Fr.; 部位:胃;	2019/10/15	1900						
A-line (1)	2	部位:左手;	2019/10/15	1900						
Penrose Drain(1)	1	號碼:10 mm.; 部位:骨盆腔;	2019/10/16	0200						

用物計數

- 手術中出血量： 150 c.c.
- 手術中尿量： 200 c.c.
-
    - 其它： 無

用物計數： 多切口同時縫合

- 1.器械： 正確，2.縫針： 正確，3.刀片： 正確，4.紗布： 正確，5.腹部墊： 未使用，6.紗球： 未使用，7.PEANUTS ： 未使用，8.紗條： 未使用，9.絲帶： 未使用，10.針頭： 未使用，11.Vessel loop： 正確，12.Boots： 未使用，13.Jelco： 未使用，14.棉條： 未使用，
    - 第一次：
        - 刷手護士 ： 羅渝軒
        - 流動護士： 上次簽章: 徐敬家
        - 時間： 0200

,

    - 第二次：
        - 刷手護士 ： 羅渝軒
        - 流動護士： 上次簽章: 徐敬家
        - 時間： 0220

,

    - 第三次：
        - 刷手護士 ： 羅渝軒
        - 流動護士： 上次簽章: 徐敬家

- 時間： 0230

備註

備註： NIL

- 記錄者： 上次簽章: 許臺真
- 時間： 2000

填寫護理人員 徐敬家

正式摘要

W95 / W95

doctorRank:B
